# Development of a Core Set of Patient-Reported Outcomes for Population-Based Cancer Survivorship Research: Protocol for an Australian Consensus Study

**DOI:** 10.2196/14544

**Published:** 2020-01-28

**Authors:** Imogen Ramsey, Nadia Corsini, Amanda D Hutchinson, Julie Marker, Marion Eckert

**Affiliations:** 1 Rosemary Bryant AO Research Centre School of Nursing and Midwifery and UniSA Cancer Research Institute University of South Australia Adelaide Australia; 2 School of Psychology, Social Work and Social Policy University of South Australia Adelaide Australia; 3 Cancer Voices South Australia Adelaide Australia

**Keywords:** cancer survivorship, quality of life, patient-reported outcomes, core outcome set, Delphi study, consensus

## Abstract

**Background:**

Core outcome sets seek to improve the consistency and quality of research by providing agreed-upon recommendations regarding what outcomes should be measured as a minimum for a population and setting. The problems arising from a lack of outcome standardization in population-based cancer survivorship research indicate the need for agreement on a core set of patient-reported outcomes (PROs) to enhance data quality, consistency, and comparability.

**Objective:**

This study aims to identify a core set of PROs, representing the most important issues impacting on cancer survivors' long-term health, functioning and quality of life, to inform population-based research on cancer survivorship.

**Methods:**

In Phase I, a list of all potentially important outcomes will be generated through focus group discussions with cancer survivors and a review of measures for assessing quality of life in cancer survivorship. The consolidated list will be advanced to Phase II, where a stakeholder consensus process will be conducted with national experts in cancer survivorship to refine and prioritize the outcomes into a core outcome set. The process will consist of a two-round Delphi survey and a consensus meeting. Cancer survivors, oncology health care professionals, and potential end users of the core outcome set with expertise in cancer survivorship research or policy will be invited to participate. In Phase III, recommended measures for assessment of the core outcome set will be selected with advice from experts on the assessment, analysis, and interpretation of PROs.

**Results:**

As of April 2019, data collection for Phase I is complete and data analysis is underway. These data will inform the list of outcomes to be advanced into Phase II. Recruitment for Phase II will commence in June 2019, and it is anticipated that it will take 6 months to complete the three-step consensus process and identify a provisional core outcome set. The study results are expected to be published in early 2020.

**Conclusions:**

Expert consensus-driven recommendations on outcome measurement will facilitate the inclusion of survivorship outcomes considered important by cancer survivors and health professionals in future research. Adoption of the core outcome set will enable comparison and synthesis of evidence across studies and enhance the quality of PRO data collected in cancer survivorship research, particularly when applied to address macro-level questions.

**International Registered Report Identifier (IRRID):**

DERR1-10.2196/14544

## Introduction

Due to the increasing incidence of cancer and advancements in its detection and treatment, the number of people living with cancer as a chronic condition is increasing [1,2]. With improved survival comes new challenges, as secondary health problems and symptoms related to cancer and treatment can persist for years following diagnosis [3]. Adverse effects including functional and cognitive impairments, physical symptoms, risk of secondary cancers, comorbidities, and poor psychosocial outcomes can have debilitating and lifelong consequences for cancer survivors and their families [3]. Accordingly, the definition of treatment efficacy has moved beyond survival alone, and there is a greater emphasis on establishing the impact of treatments on quality of life [4]. Increasing efforts to quantify the subjective experience of illness, treatment, and side effects using patient-reported outcomes (PROs) have led to a proliferation in the number of patient-reported outcome measures (PROMs) available to assess aspects of health, functioning, and quality of life from the patient's perspective [4].

The potential uses of PRO data can be understood using the Lipscomb framework for cancer outcomes research, which proposes three arenas for the application of PROs: micro, meso, and macro [5]. At the micro or individual level, PRO data are used to support and enhance patient-centered care, patient-clinician interactions, and decision making in clinical settings [4]. At the meso or service level, PRO data are used to understand the variables that influence health outcomes. PRO data collected at a macro level can be used to monitor and understand trends in health outcomes across a population [5]. Although there are few established systems internationally for the routine collection of macro-level PRO data (known as PRO surveillance), there is increasing recognition that expanding and optimizing the role of cancer registries to include PRO surveillance would improve our understanding of the long-term trajectory of survivorship outcomes on a national scale [6].

A challenge in PRO research is the lack of standardization and comparability of scores from different PROMs. For any given outcome, there may be multiple measures developed for different purposes, populations, and disciplines. Core outcome sets have been proposed as a way of addressing these problems. A core outcome set is a recommended, standardized, and minimum set of outcomes to be measured and reported in research on a specific health condition and which should be agreed upon by relevant stakeholders [7,8]. By standardizing the outcomes that are examined across studies, use of core outcome sets can help minimize bias in outcome selection and reporting and facilitate data comparison and synthesis [9]. Core outcome sets have been developed for various diseases including cancer, but not for the assessment of long-term cancer survivorship concerns at a population level. Predominantly tumor-specific core outcome sets with a typically acute focus have been developed to facilitate standardized assessment of treatment-related symptoms and toxicities in clinical trials.

This study aims to develop a core outcome set, recommending what PROs should be collected as a minimum for surveillance of cancer survivorship concerns in Australia and how these outcomes should be measured. The need for a set of outcomes applicable to all cancers is underscored by research identifying issues common across cancer sites in the long-term [10] including psychosocial outcomes, fatigue, functional impairment, fear of recurrence, and limitations in health care and insurance access [11]. The core outcome set will be developed using recommended methods, including a Delphi consensus process. The Delphi technique is a well-recognized method for obtaining expert consensus on a defined problem [12] and is used frequently in health research and core outcome set development.

## Methods

### Study Design

The core outcome set will be developed using a comprehensive, stepwise approach based on recommendations for core outcome set development [7] and previous studies [13,14]. This approach draws on the lived experiences of cancer survivors, published literature, and expert opinion. The study design was informed by the Core Outcomes Measurement in Effectiveness Trials (COMET) initiative and the Outcome Measures in Rheumatology (OMERACT) initiative, which provide methodological guidance to support evidence-based core outcome set development [15,16]. The OMERACT framework was developed in rheumatology and proposes a recommended process for core outcome set development that can be applied to different health conditions [16,17]. Both initiatives agree that the development of a core outcome set requires consensus to be reached first on “what” to measure (ie, the core outcomes) and then on “how” to measure the outcomes (ie, the measures) [7]. Therefore, a list of recommended outcomes to be measured will be one deliverable independent of the recommended PROM(s) for assessing the outcomes. Given that PROMs are continually researched and improved, this will allow for periodical revision of the recommended measures, given new advances. The Core Outcome Set-Standards for Reporting (COS-STAR) statement will be used to demonstrate transparency and complete reporting. The statement comprises an 18-item checklist covering the minimum reporting requirements for the sections of a paper describing the development of a core outcome set [18].

The research will involve three phases:

- Phase I: Identifying all possible outcomes relevant to cancer survivorship via focus groups with cancer survivors and a review of available PROMs.
- Phase II: Reaching expert consensus on the most important outcomes for inclusion in the core outcome set.
- Phase III: Determining how to best measure the core outcomes.

This study has been approved by the University of South Australia Human Research Ethics Committee (application number: 200370)

### Phase I: Generating a List of Possible Outcomes

In Phase I, a list of possible outcomes will be generated via two processes: (1) focus groups with cancer survivors and (2) a review of cancer survivorship PROMs.

#### Focus Groups with Cancer Survivors

##### Aims and Scope

Focus groups will be conducted with adult cancer survivors to explore their experiences of living with cancer and its impacts on physical, functional, social, and psychological health and wellbeing. Five focus groups of 8-10 participants will be held with cancer survivors in Adelaide and Sydney.

##### Participants

Adult cancer survivors who are over 18 years of age, English speaking, have been diagnosed with any type of cancer, and are not currently undergoing primary treatment will be eligible to participate. A diverse sample of participants in terms of age, geographical location and cancer type will be sought to ensure that a range of views are considered. Participants will be recruited via the networks of the University of South Australia and state and national cancer organizations. The study will be advertised on social media, the University of South Australia Cancer Research Institute volunteers website, and flyers posted at the University of South Australia

##### Format

The focus group discussions will be guided by open-ended questions about the long-term impacts of cancer and treatment on everyday life.

##### Data Analysis

A list of outcomes derived from the transcribed focus group discussions will be generated and organized into a conceptual PRO framework. The outcomes will be periodically reviewed and modified, where necessary, to ensure fit with the data.

#### Review of Cancer Survivorship Patient-Reported Outcome Measures

##### Aims and Scope

To determine the outcomes considered relevant in cancer survivorship research, an updated review of PROMs developed to assess quality of life in long-term cancer survivors will be conducted. In concordance with previous studies, long-term cancer survivors will refer to people who have been diagnosed with cancer, are not currently receiving primary treatment, and are likely facing challenges and symptoms unique to those experienced by cancer patients under treatment and the general population [19].

##### Inclusion Criteria

English-language papers describing the characteristics of PROMs developed to assess quality of life in long-term adult cancer survivors will be included. PROMs developed specifically for pediatric cancer survivors will be excluded.

##### Data Sources

Searches for peer-reviewed articles will be performed in Medline, EMBASE, CINAHL, Scopus, the Joanna Briggs Institute Database of Systematic Reviews, and the Cochrane Library. Indexing terms and subject headings for each database will be added.

##### Data Extraction

Identified articles will be classified based on the PROMs they refer to. For each measure, the intended population, number of items, included dimensions or constructs, study sample, scoring information, and tested validity and reliability will be extracted. A list of outcome domains will be extracted from the identified instruments and examined for overlap in content and terminology, consistent with the approach taken by Macefield et al [9] for esophageal cancer.

##### List Consolidation

The list of outcome domains will be used in Round 1 of the consensus process. To consolidate the list, the outcome domains derived from the focus groups and review will be combined and examined by the research team to ensure that there is no duplication and that definitions are clear. The following three criteria adapted from Reeve et al [10] will be required for inclusion of an outcome domain: (1) present across diverse cancer types, (2) attributable to cancer or treatment, and (3) amenable to patient self-report. Additionally, it is proposed that outcomes must be appropriate for the intended end use of PRO surveillance.

### Phase II: Consensus Process

#### Delphi Method

The consensus process will incorporate a three-step modified Delphi method. The Delphi method involves administering sequential surveys anonymously to a group of selected experts. The process traditionally begins with open-ended questions that are subsequently refined through a series of rounds, interspersed with controlled feedback based on the group views, until consensus is reached [7,12,20]. This study will use a modified Delphi method, limited to two survey rounds and a final consensus meeting. The modifications to the Delphi method are beginning the process with purposely selected (rather than open-ended) questions and including a final face-to-face meeting. Key advantages of preselecting items are that it provides a solid grounding in existing evidence and can improve response rates [20]. Benefits of including a face-to-face meeting are that it allows panel members to interact and discuss, clarify, and justify their voting [21]. This approach has been favored over other consensus methods by participants and is perceived as highly cooperative and effective [22]. Both modifications are consistent with recommended methods for evidence-based core outcome set development [23].

#### Aims and Scope

To refine the list of outcomes identified in Phase I into a cancer survivorship core outcome set, key stakeholders will be invited to participate in an online Delphi survey. The list of outcome domains will be formatted into questionnaire items and participants will be asked to rate the importance of each item for inclusion in the core outcome set, with higher scores indicating higher importance.

#### Participants

Recent methodological work has demonstrated the importance of receiving feedback from different groups of stakeholders in order to reach consensus on a core outcome set [8]. Therefore, three expert panels of approximately 25 participants will be recruited for the study: (1) adult cancer survivors, (2) clinicians (ie, physicians, nurse practitioners, and clinical nurse specialists with expertise caring for and treating people with cancer), and (3) other health care professionals (ie, allied health workers, psycho-oncologists, nurses) and potential end users of the core outcome set that have cancer survivorship expertise (ie, researchers, policy advisors).

#### Recruitment

As it is common practice to purposefully select experts to participate in a Delphi process, health and research professionals with cancer survivorship expertise will be identified via professional networks. Focus group participants will be invited to participate and, if necessary, additional cancer survivors will be identified via the same recruitment channels as the focus group participants. All identified stakeholders will receive an email outlining the aims and significance of the research, with a link to register their interest in participating. Online registration will be open for 4 weeks, and two reminder emails will be sent. Written consent will be obtained from the study participants.

#### Round 1

All registered stakeholders will receive an email detailing the study purpose, a participant information sheet, and a link to the online survey for Round 1. The participant information sheet will explain the study background and objectives, intended end use of the core outcome set, key definitions, and ethical considerations in lay terms. The survey will contain questions about participants’ demographics and expertise in cancer survivorship and the list of outcome domains formatted into items. Participants will be asked to rate the importance of each item for inclusion in a core outcome set for surveillance of the long-term impacts of cancer on quality of life using the 9-point Grading of Recommendations Assessment, Development and Evaluation (GRADE) scale for the quality of evidence of outcomes. The GRADE scale has been previously used in the development of core outcome sets involving the Delphi technique [14,24]. Scores of 7-9 indicate critical importance, scores of 4-6 indicate importance, and scores of 1-3 indicate limited importance. A blank text response option will be provided for participants to add nonlisted outcomes and explain their rankings if they wish to. Nonresponders will receive up to three reminders to participate.

#### Feedback and Round 2

Score allocations from Round 1, for each panel and overall, will be presented to participants along with the link to the Round 2 survey. A recent trial found that the type of feedback given to participants (ie, whether they received a summary of the voting for their own group, all groups, or all participants regardless of group) did not influence voting [25]. After receiving feedback, participants will be invited to rescore the outcomes from Round 1 as well as any additional outcomes suggested.

#### Consensus Definition

Items with a median score ≥7 and an interquartile range no larger than two units, that receive rankings of 7-9 by ≥70% of participants and 1-3 by ≤15% of participants after Round 2 will be eligible for inclusion in a provisional core outcome set and progressed to the consensus meeting. There are no universally agreed-upon consensus criteria in Delphi studies; these thresholds follow published recommendations and previous core outcome set development studies in cancer [23,26]. To ensure that outcomes considered important to individual stakeholder groups are not rejected without the opportunity for reflection, items for which consensus is ambiguous will also be discussed in the consensus meeting. Consensus will be deemed ambiguous if an outcome with a median score ≥7 has an interquartile range no larger than three units and ≥65% majority agreement for at least one panel.

#### Analysis of Voting

Descriptive statistics will be used to summarize the results for each round, including the median score for each item and percentage of ratings between 1-3, 4-6, and 7-9. The results will be presented for each panel and overall. A measure of the distribution of scores will be presented for each outcome domain considered in Round 2. This is recommended because major disagreement can be masked by cut-off scores, which do not capture the strength of minority opinion [27].

#### Consensus Meeting

After Round 2, 10-15 Delphi participants will meet to review, discuss, and agree on a final set of core outcome domains. The participants will be purposively sampled from those who completed both survey rounds to ensure a range of perspectives are represented.

### Phase III: Instrument Appraisal and Selection

After reaching consensus on the core outcome domains, the final step will be to identify and select measures for their assessment. The PROMs identified in the review will be evaluated by applying the OMERACT Filter 2.0, a tool for selecting measures that summarizes three key measurement properties: truth, discrimination, and feasibility [17]. Truth refers to face, content, and construct validity; discrimination captures reliability and sensitivity to change; and feasibility refers to whether a measure can be applied in the intended setting, given the constraints of time, money, and interpretability [17]. The final selection of PROMs will be undertaken through consultation with the Quality of Life Office, which is a national resource funded by Cancer Australia that provides expert advice related to the design, assessment, analysis, and interpretation of PROs. Feedback on the final core outcome measurement set will be sought from the Delphi participants via an informal process.

## Results

As of April 2019, data collection for Phase I is complete and data analysis is underway. These data will inform the list of outcomes to be progressed into Phase II. Recruitment for Phase II will commence in June 2019, and it is anticipated that it will take 6 months to complete the three-step consensus process and identify a provisional core outcome set. The study results are expected to be published in early 2020.

## Discussion

Problems arising from the heterogeneity of PROMs have been documented in cancer survivorship research [28] and other areas of health research [15]. A review of registries that systematically collect PRO for population-level surveillance of cancer survivorship outcomes recommended that consensus be obtained on a core set of outcome domains and measures to improve the consistency and comparability of data collected [28]. Adoption of the proposed core outcome set by researchers could enhance the quality of future research by helping ensure the inclusion of relevant survivorship outcomes that are important to consumers and the use of validated tools that are sensitive to survivorship. In addition to macro-level application, the proposed core outcome set could also guide researchers seeking to use survivorship PROMs in clinical trials or extended follow-up beyond acute settings. To maintain relevance, the core outcome set may need to be periodically checked and updated to reflect consequences of new treatment modalities.

This is not the first core set of outcomes to be developed for all cancers, although it is unique in its scope and intended end use. In 2011, the National Cancer Institute’s Symptom Management and Health-Related Quality of Life Steering Committee led an international effort to identify a core set of patient-reported symptoms to be measured in all adult cancer treatment trials [10] as well as disease-specific core symptom sets for head and neck, prostate, and ovarian cancers [29-31] to be used in conjunction with the generic cancer set. Since self-reported symptoms offer important insight into intervention efficacy and toxicities, increased consistency of symptom assessment across trials using the patient-reported core symptom sets may help improve care quality for clinical trial participants [10]. Our study aims to promote consistent assessment across the growing number of systems collecting PRO data from cancer survivors at a population level. Therefore, agreement on a core set of PROs similar to the set developed by the National Cancer Institute but (1) representing the health, functioning, and quality of life issues that are important in long-term cancer survivorship (ie, not exclusive to disease and treatment-related symptoms) and (2) suitable for the purpose of macro-level surveillance (ie, not specific to clinical trials) is warranted [10,28]. Despite a shared aim of facilitating consistent assessment of PROs in research with cancer populations [10], the core outcome set developed by the National Cancer Institute will inform assessment of disease- and treatment-related symptoms in clinical trials, while the proposed core outcome set for cancer survivorship will play a complementary role in informing population-based surveillance of quality of life among cancer survivors beyond clinical settings.

In the Netherlands, Geerse et al [32] developed a core set of International Classification of Functioning, Disability, and Health (ICF) categories for health-related problems in adult cancer survivors. In addition to being based on the ICF, their Cancer Survivor Core Set differs from the proposed core outcome set in intended purpose and development methods. Three panels of 25 survivorship experts for colorectal, breast, and lung cancers participated in a two-round Delphi study where 265 ICF categories were used for item selection [32]. The resulting core outcome set included 19 items, 11 of which were linked to corresponding item(s) on at least one of three selected cancer survivorship PROMs. The intended application of the core outcome set developed by Geerse et al [32] is not specified, although the authors state that it could be used as a screening tool. In contrast, the proposed core outcome set is intended to reflect the most important and relevant survivorship issues beyond acute symptoms that should be collected in routine PRO surveillance in Australia. Item selection will be informed by existing PROMs and oncological PRO frameworks rather than the ICF. Adults of different ages with a diverse range of cancers will be represented in the development process. It will be beneficial to understand the similarities and differences in outcomes prioritized by the current Australian-led research and the core outcome set developed in the Netherlands.

We based the study methods on recommendations for evidence-based core outcome set development [23], guidelines for using the Delphi technique to obtain consensus on core outcomes [27], standards for core outcome set study design [33], and previous core outcome set studies with cancer populations that employed Delphi methods [26,34]. However, there is no agreed-upon methodology for developing a core outcome set. It is therefore unclear to what extent the results from this study would be concordant with those obtained in different settings, using alternate consensus methods, or applying different criteria. Despite these limitations, the study design is considered suitable for the scope and setting of this core outcome set and will allow a large and geographically diverse sample of stakeholders to participate.

Although there is little scientific evidence regarding the optimal number of Delphi rounds, two or three rounds have been frequently recommended [35] and commonly used in core outcome set development studies [23]. Given that we will undertake a rigorous process to identify, select, and refine the initial outcome list to be progressed into the Delphi and include a face-to-face meeting to agree on the final core outcomes, two survey rounds were considered sufficient. An advantage of restricting the number of rounds is that this can limit potential bias due to attrition, which is likely to increase with each round. A limitation of restricting the study to two survey rounds is that it may not be possible to confirm stability of voting, although this is generally thought to be a measure of internal reliability and not consensus. Instead, we will be measuring the extent to which participants agree with the statements under consideration (agreement) and the extent to which participants agree with each other (consensus) [36]. It is not possible to determine the validity of any specific definition of consensus in Delphi studies, but the proportion of ratings within a range is one of the most commonly employed consensus definitions and the median is considered the most robust measure of central tendency [36].

Another potential limitation is that we are conducting a review of cancer survivorship PROMs rather than of all outcomes that have been examined in cancer survivorship research, and therefore, we may not identify all possible relevant outcomes. This risk will be mitigated by additionally identifying outcomes in Phase I from focus groups with cancer survivors and providing Delphi participants with the opportunity to suggest additional outcomes in Phase II.

This protocol describes the development methods for a core set of PROs to inform our understanding of the long-term impacts of cancer on survivors’ quality of life at a population level. Since the proposed set represents the minimum outcomes that should be collected and reported on, it can be supplemented with other outcomes or measures relevant to a given study setting or population. By providing consensus-driven recommendations from stakeholders with expertise in cancer survivorship research, practice, policy, and lived experience, the study findings will facilitate the inclusion of meaningful survivorship outcomes and enhance the quality and comparability of PRO data collected in survivorship research, particularly when applied to address macro-level questions.

## Abbreviations

COMET: Core Outcome Measures in Effectiveness Trials
COS-STAR: Core Outcome Set-Standards for Reporting
GRADE: Grading of Recommendations Assessment, Development and Evaluation
ICF: International Classification of Functioning, Disability and Health
OMERACT: Outcome Measures in Rheumatology
PRO: patient-reported outcome
PROM: patient-reported outcome measure
